# Association of American Medical Editors—Annual Meeting, May 6, 1872

**Published:** 1872-05-01

**Authors:** F. H. Davis

**Affiliations:** Secretary, Per N. S. D.


### ASSOCIATION OF AMERICAN MEDICAL EDITORS—ANNUAL MEETING, MAY 6, 1872.

The members of the Association assembled in the committee room of Horticultural Hall, Broad street, Philadelphia, and were called to order at 11 o'clock a. m.

The President, Dr. B. F. Dawson of New York in the chair.

The following members were present: Dr. B. F. Dawson, of "*The American Journal of Obstetrics and Diseases of Women;* Dr. S. W. Butler, of *The Medical and Surgical Reporter;* Dr. Stone, of *The Northwestern Medical and Surgical Journal;* Drs. D. W. Yandell and Theophilus Parvin, of *The American Practitioner*, and Dr. N. S. Davis, of THE CHICAGO MEDICAL EXAMINER.

The acting Secretary, Dr. N. S. Davis of Chicago, read the Articles of Association and the Records of Proceedings of the last Annual Meeting.

On motion of Dr. A. G. Stone of St. Paul, the Secretary was requested to obtain from the former President, Dr. H. R. Storer of Boston, the names of such editors as joined the Association during his term of office and authorized those names to be appended to the constitution, that they may be properly enrolled on the list.

Dr. T. Parvin, of Indianapolis, Chairman of Committee on Foreign Exchanges, reported recommending the acceptance of the offer made by Prof. Henry, as stated in the minutes of the last annual meeting, and asking that the committee be discharged. After some remarks by Dr. Dawson, also a member of the committee, the report was accepted and the request of the committee for a discharge, granted.

Dr. Dawson, suggested the propriety of offering an Annual Prize on some subject to be determined by the Association as a means of advancing the cause of science and the efficiency of the Association. He offered to contribute $100 for that purpose. Drs. N. S. Davis, D. W. Yandell, T. Parvin, and A. G. Stone, each offered to contribute a like sum. Whereupon the following resolution was unanimously adopted:

*Resolved*, That an annual prize of $100 be offered by this Association for the best essay, on some subject to be decided upon at each annual meeting, and the same to be open for competition to all medical editors belonging to this Association.

In accordance with this resolution, Drs. Dawson, Davis and Stone were appointed a committee to recommend a subject for the prize essays of the coming year, and to nominate a committee to examine the same.

The Secretary called attention to an error in the record of proceedings of the first meeting of the Association, by which the name of Dr. Dawson was omitted, who was present and participated in the doings of that meeting. On motion the Secretary was authorized to correct the minutes by recording the name of Dr. B. F. Dawson as a member of the meeting organizing the Association in New Orleans, La.

On motion the Association adjourned until 8 o'clock p. m.

EVENING SESSION.

Association was called to order at 8½ p. m., the President, Dr. Dawson, in the chair.

Dr. A. B. Palmer, of the *University Medical Journal* of Michigan, took his seat. The President then delivered an interesting address on the Origin of Medical Science, referring chiefly to the period anterior to the time of Hippocrates.

The committee appointed on a proper subject for prize essays recommended the following for the prize to be awarded in May 1873:

"The Pathology and Treatment of Diseases of the Ovaries."

And for the prize to be awarded in May 1874, the following:

"At what stages of Pulmonary Tuberculosis is a change of climate desirable; what are the principles which should govern us in choosing the kind of change to be made; and the best localities in North America to send patients of this class?"

The report of the committee was accepted and the recommendation adopted.

On motion, Drs. A. B. Palmer of Ann Arbor, Michigan, B. F. Dawson of New York, and Brinton of Philadelphia, were appointed a committee to award the prize of 1873, to be governed in their action by the same rules that govern the committee on prize essays of the American Medical Association; all essays designed for the prize, to be sent to the chairman of the committee on or before the first of March 1873.

On motion the Secretary was instructed to have published in the various medical journals, the subjects for prize essays, and the regulations just adopted concerning them.

The following gentlemen were then elected officers for the ensuing year:

President, Dr. Theophilus Parvin of Indianapolis; Vice-President, Dr A. G. Stone of St. Paul; Secretary, Dr. F. H. Davis of Chicago.

On motion of Dr. Davis, the thanks of the Association were tendered to the retiring president, for the faithful discharge of his duties, and a copy of his address requested for publication.

On motion the Secretary was instructed to call the next annual meeting at 8 o'clock in the evening of Monday preceding the first Tuesday in May 1873.

There being no further business the Association adjourned.

F. H. Davis, *Secretary*.
Per N. S. D.

Reproduction of the Tibia.—Dr. Cheever reports the removal of the entire shaft and lower epiphysis of the tibia, with its subsequent reproduction, a useful limb resulting. Dr. Cheever appends to his article extracts from five other similar cases.—*Richmond and Louisville Med. Jour.*

Carbolic Acid as a Local Anæsthetic.—If a portion of skin is covered with a cloth soaked in a saturated solution of carbolic acid for half an hour, and then a streak traced across the surface with a camel's hair pencil diffused in acid liquefied by one-twentieth its bulk of water, the skin may be divided along the course of the streak with a sharp scalpel, quite down to the subcutaneous tissue, without pain. Abscesses, whitloes and buboes may be opened in this way with great advantage.—*Atlanta Med. and Surg. Jour.*

